# The Connexin50D47A Mutant Causes Cataracts by Calcium Precipitation

**DOI:** 10.1167/iovs.18-26459

**Published:** 2019-05

**Authors:** Viviana M. Berthoud, Junyuan Gao, Peter J. Minogue, Oscar Jara, Richard T. Mathias, Eric C. Beyer

**Affiliations:** 1 Department of Pediatrics, University of Chicago, Chicago, Illinois, United States; 2 Department of Physiology and Biophysics, Stony Brook University, Stony Brook, New York, United States

**Keywords:** calcium precipitation, connexin50, cataract, lens circulation

## Abstract

**Purpose:**

Mutations in connexin50 (Cx50) and connexin46 (Cx46) cause cataracts. Because the expression of Cx46fs380 leads to decreased gap junctional coupling and formation of calcium precipitates, we studied Cx50D47A lenses to test whether Cx50 mutants also cause cataracts due to calcium precipitation.

**Methods:**

Connexin levels were determined by immunoblotting. Gap junctional coupling conductance was calculated from intracellular impedance studies of intact lenses. Intracellular hydrostatic pressure was measured using a microelectrode/manometer system. Intracellular free calcium ion concentrations ([Ca^2+^]_i_) were measured using Fura-2 and fluorescence imaging. Calcium precipitation was assessed by Alizarin red staining and compared to the distribution of opacities in darkfield images.

**Results:**

In Cx50D47A lenses, Cx50 levels were 11% (heterozygotes) and 1.2% (homozygotes), and Cx46 levels were 52% (heterozygotes) and 30% (homozygotes) when compared to wild-type at 2.5 months. Gap junctional coupling in differentiating fibers of Cx50D47A lenses was 49% (heterozygotes) and 29% (homozygotes), and in mature fibers, it was 24% (heterozygotes) and 4% (homozygotes) compared to wild-type lenses. Hydrostatic pressure was significantly increased in Cx50D47A lenses. [Ca^2+^]_i_ was significantly increased in Cx50D47A lenses. Alizarin red-stained calcium precipitates were present in homozygous Cx50D47A lenses with a similar distribution to the cataracts.

**Conclusions:**

Cx50D47A expression altered the lens internal circulation by decreasing connexin levels and gap junctional coupling. Reduced water and ion outflow through gap junctions increased the gradients of intracellular hydrostatic pressure and concentrations of free calcium ions. In these lenses, calcium ions accumulated, precipitated, and formed cataracts. These results suggest that mutant lens fiber connexins lead to calcium precipitates, which may cause cataracts.

Cataracts are opacities in the lens that disrupt the normal transmission and focusing of light onto the retina. They can be caused by a variety of diseases and environmental factors. They are often associated with aging. Congenital cataracts are caused by mutations of a number of genes expressed in the lens (summarized in http://cat-map.wustl.edu/).^1^ Cataracts may form due to disruption of lens homeostasis. Because the lens is avascular, it maintains its transparency and homeostasis through an internal circulation system.^2^ Many types of cataracts may result from disturbances of the lens circulation.

The lens circulation system allows flow of water, ions and small molecules from the surface of the lens to its center, driven by ion pumps, exchangers, and transporters and back to the surface by gap junction-mediated intercellular communication (reviewed by Mathias et al.).^2^ Gap junctions contain channels that allow the passive passage of cytoplasmic ions and small molecules directly from cell to cell. They are formed by the coaxial alignment of two hemichannels, each contributed by one of the cells that form the junction. Each hemichannel is a hexameric assembly of protein subunits called “connexins.” Connexin46 (Cx46) and connexin50 (Cx50) form the gap junctions between lens fiber cells.^3^,^4^

Alterations of lens fiber connexins have been implicated in cataract formation. Mice with targeted deletion of Cx46 or Cx50 develop cataracts.^5^,^6^ Many non-functional Cx46 and Cx50 mutants have been linked to human congenital cataracts (reviewed by Beyer et al., Berthoud and Ngezahayo).^7^,^8^ We have been using mouse models that mimic some of these mutations to gain insight into how they lead to cataracts. We recently showed that Cx46fs380 lenses have decreased gap junctional coupling, leading to calcium accumulation and formation of precipitates.^9^,^10^ This raised the question whether expression of Cx50 mutants led to a similar process in which the lens circulation is disrupted and Ca^2+^ accumulates. Therefore, we used lenses that express the non-functional cataract-causing Cx50 mutant, Cx50D47A,^11^–^13^ to characterize the effect of this mutation on the lens circulation and Ca^2+^ levels.

## Materials and Methods

### Animals

Experiments were performed on Cx50D47A mice (originally identified by Favor by screening for the cataract phenotype following ethylnitrosourea mutagenesis)^11^ that were maintained on the original C3H genetic background.^13^

All animal procedures were performed in accordance with the ARVO Statement for the Use of Animals in Ophthalmic and Vision Research. Protocols were approved by the Institutional Animal Care and Use Committees of the University of Chicago and Stony Brook University.

### Determination of Gap Junctional Coupling, Intracellular Hydrostatic Pressures, and Calcium Ion Concentrations

The gap junction coupling conductances, hydrostatic pressures, and concentrations of calcium ions were determined in wild-type and Cx50D47A heterozygous and homozygous lenses as previously described.^10^,^14^–^16^ The age range of the animals was 60 to 77 days for intracellular pressure studies, 81 to 97 days for impedance determinations, and 101 to 117 days for intracellular calcium ion concentration. All experiments were conducted on freshly dissected lenses. Data from at least eight lenses of the same genotype were pooled to quantify the radial gradients of series resistance, hydrostatic pressure, and calcium ion concentration. The data presented in the graphs include all experimental measurements. The curves in the graphs represent the best-fit of the data to the models that describe changes in these parameters as a function of relative distance from the lens center.^10^,^14^–^16^

The gap junction coupling conductance per area of radial contact between fiber cells was calculated from the reciprocal of resistivity multiplied by fiber cell width:

$$\newcommand{\bialpha}{\boldsymbol{\alpha}}\newcommand{\bibeta}{\boldsymbol{\beta}}\newcommand{\bigamma}{\boldsymbol{\gamma}}\newcommand{\bidelta}{\boldsymbol{\delta}}\newcommand{\bivarepsilon}{\boldsymbol{\varepsilon}}\newcommand{\bizeta}{\boldsymbol{\zeta}}\newcommand{\bieta}{\boldsymbol{\eta}}\newcommand{\bitheta}{\boldsymbol{\theta}}\newcommand{\biiota}{\boldsymbol{\iota}}\newcommand{\bikappa}{\boldsymbol{\kappa}}\newcommand{\bilambda}{\boldsymbol{\lambda}}\newcommand{\bimu}{\boldsymbol{\mu}}\newcommand{\binu}{\boldsymbol{\nu}}\newcommand{\bixi}{\boldsymbol{\xi}}\newcommand{\biomicron}{\boldsymbol{\micron}}\newcommand{\bipi}{\boldsymbol{\pi}}\newcommand{\birho}{\boldsymbol{\rho}}\newcommand{\bisigma}{\boldsymbol{\sigma}}\newcommand{\bitau}{\boldsymbol{\tau}}\newcommand{\biupsilon}{\boldsymbol{\upsilon}}\newcommand{\biphi}{\boldsymbol{\phi}}\newcommand{\bichi}{\boldsymbol{\chi}}\newcommand{\bipsi}{\boldsymbol{\psi}}\newcommand{\biomega}{\boldsymbol{\omega}}{G_{MF,DF}} = 1/\left( {w{R_{MF,DF}}} \right)$$

where *w* corresponds to the cell width (≈ 3 μm) and R_MF_ and R_DF_ correspond to the effective intracellular resistivities of fiber cells in the central mature fiber domain (MF) and peripheral differentiating fiber domain (DF). The effective intracellular resistivities are dominated by the resistance of gap junctions; they are essentially inversely proportional to the number of open gap junction channels per area of radial cell-to-cell contact.

Intracellular hydrostatic pressure was measured using a manometer to adjust and record the pressure within an intracellular microelectrode. When intracellular and intra-microelectrode pressures are the same, the tip resistance of the microelectrode is constant at the value recorded in the external bathing solution. Pressure data from 8 to 10 lenses were pooled to obtain the pressure-depth curves shown in “Results.”

Intracellular calcium was determined by microinjecting Fura-2 into fiber cells at various depths into several lenses followed by optically recording the fluorescence emission at 360 nm and 380 nm excitation. The ratios of fluorescence emission at 360 nm/380 nm excitation were compared to a calibration curve to obtain the concentrations of free calcium ions. Data from 12 lenses were pooled to obtain the concentration-depth curves shown in “Results.”

### Immunoblotting

Lenses from mice at different ages were dissected out in PBS, pH 7.4 and homogenized in PBS containing 4 mM EDTA and cOmplete EDTA-free protease inhibitor cocktail (Roche Applied Science, Indianapolis, IN, USA). Aliquots of homogenates from wild-type and Cx50D47A heterozygous and homozygous lenses containing equal amounts of total proteins were loaded per lane. Proteins were resolved on SDS-containing polyacrylamide gels and subjected to immunoblot analysis, as previously described.^17^ Equal loading and transfer were confirmed by staining the membranes with Ponceau S before incubation with primary antibodies.^18^ Three independent experiments containing all genotypes were performed. The intensity of the bands was analyzed by densitometry using Adobe Photoshop (Adobe Systems, San Jose, CA, USA). Results from heterozygotes and homozygotes are reported as percentages of the values determined in wild-type samples. Statistical significance was assessed by using paired Student's *t*-test.

### Whole-Mount Calcium Staining

Lenses from 21-day-old wild-type and homozygous Cx50D47A mice were used for easier penetration of Alizarin red. Lenses were dissected in PBS, pH 7.4. The freshly dissected lenses were photographed under darkfield illumination. Lenses were then processed for whole-mount staining, as previously described.^10^ Lens images (before and after staining) were obtained using a Zeiss Stemi-2000C dissecting scope (Carl Zeiss Microscopy GmbH, Munich, Germany) and a Zeiss AxioCam digital camera using Zeiss AxioVision software.

### Determination of Lens Refractive Properties

To illustrate changes in the refractive properties, lenses from 33-day-old (an age when cataracts are obvious but not as pronounced as at older ages) and 108-day-old mice were photographed on top of a 200-mesh electron microscopy grid, as previously described.^9^,^19^ Lenses were viewed using a Zeiss Stemi-2000C stereo microscope (Zeiss Microscopy) equipped with a halogen lighting system for transmitted illumination and a Schott KL 1500 LCD cold light source that uses a 15 V/150 W halogen reflector lamp producing a color temperature of 2900 K. Images were acquired with a Zeiss AxioCam digital camera using Zeiss AxioVision software.

Images of lenses from wild-type C3H and 129 × C57BL/6J mice (80–84 days old) were obtained as described above. In these lenses, the warping index was determined at different distances from the lens center using the bUnwarp plug-in from ImageJ^20^,^21^ (http://imagej.nih.gov/ij/; provided in the public domain by the National Institutes of Health, Bethesda, MD, USA).

## Results

### Gap Junction Coupling Conductance Is Decreased in Cx50D47A Lenses

To assess the effect of Cx50D47A expression on gap junction coupling conductance, the series resistance was measured in wild-type (+/+), heterozygous (+/Cx50D47A) and homozygous (Cx50D47A/Cx50D47A) lenses.

**Figure 1:**
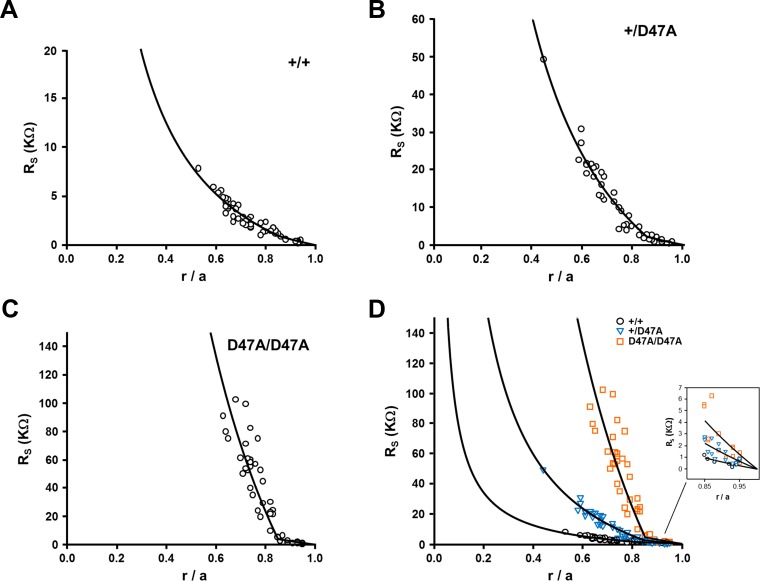
Lenses from Cx50D47A mice have decreased gap junctional conductance. (A−C) Graphs show the series resistance (R_s_) due to gap junctions coupling fiber cells between the point of recording and the surface of the lens. Data from wild-type (A), heterozygous mutant (B), and homozygous mutant (C) lenses are graphed as a function of radial distance from the lens center (r; cm), normalized to the lens radius (a; cm). The effective resistivities, R_DF_ and R_MF_, were determined by fitting the data to the model described in Mathias et al.^14^ (D) A graphical comparison of the data from the three types of lenses. The inset shows an expanded view of the data in the differentiating fiber cells (r/a ≥ 0.85).

The lens series resistance is the radial resistance of all the gap junctions between the point of recording and the surface of the lens. It showed a progressive increase from wild-type to heterozygous to homozygous Cx50D47A mice (Fig. 1). This is a function of the effective intracellular resistivities (R_MF_ and R_DF_) of fiber cells in the central mature fiber domain (MF) and peripheral differentiating fiber domain (DF). The increase in series resistance from wild-type lenses to heterozygous to homozygous mutant lenses indicates a progressive loss of gap junctional coupling that suggests fewer open channels per area of cell-to-cell contact. Lens coupling conductance in differentiating fibers decreased to 49% in Cx50D47A heterozygotes and to 29% in homozygotes compared to the wild-type values (Table 1). The decrease in mature fibers was more pronounced: gap junctional coupling was decreased to 24% of the wild-type values in Cx50D47A heterozygotes and to 4% of the wild-type values in Cx50D47A homozygotes (Table 1). Interestingly, gap junctional coupling in wild-type lenses from the C3H background was about half that determined in wild-type lenses from the 129 × C57BL/6J mixed background (Table 1).

**Table 1:** Gap Junctional Coupling Conductance in Wild-Type and Cx50D47A and Cx50-Null Lenses

**Genotype**	**a (cm)**	**G_DF_ (S/cm^2^)**	**G_MF_ (S/cm^2^)**	***n***
+/+	0.121	0.41	0.25	8
+/Cx50D47A	0.105	0.20	0.06	8
Cx50D47A/Cx50D47A	0.096	0.12	0.01	8
Cx50^+/+^	0.101	0.95	0.44	–
Cx50^+/−^	0.101	0.63	0.45	–
Cx50^−/−^	0.080	0.41	0.14	–

Table 1 shows the radii (a) and cell-to-cell gap junctional coupling conductance per unit area of radial contact between fiber cells in wild-type and Cx50D47A lenses (present study) and of wild-type and Cx50-null lenses (Baldo et al.^30^). The Cx50D47A mice were maintained on the C3H genetic background, and the Cx50-null mice were maintained on a 129/Sv × C57BL/6J mixed genetic background. The differentiating fiber (DF) and mature fiber (MF) radial coupling conductances per area of cell-to-cell contact were calculated from *G_DF,MF_* = 1/*wR_DF,MF_*, where the fiber cell width, *w*, is assumed to be 3 μm.

The lens nucleus from these mice (on the C3H genetic background) was unusually hard and difficult to impale with a microelectrode without clogging the tip. Consequently, the series resistance data were only recorded in fiber cells less than half the distance into the center of the lens (Fig. 1). The impalement problem was worse in the heterozygous and homozygous Cx50D47A lenses than in wild-type lenses (Fig. 1). Series resistance at the center of the lens was extrapolated from the best fit of the model curve to fairly peripheral mature fiber cells (Fig. 1).

### Cx46 and Cx50 Levels Are Reduced in Cx50D47A Lenses

**Figure 2:**
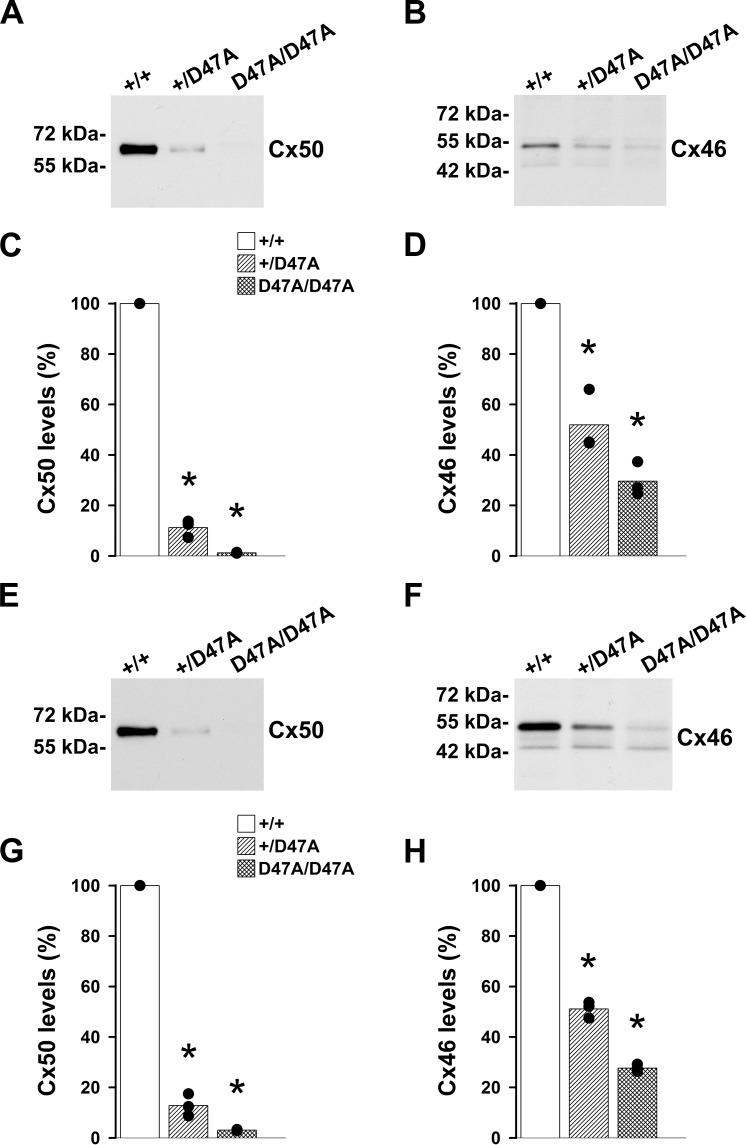
Expression of Cx50D47A decreases levels of Cx46 and Cx50. (A, B) Immunoblots show the Cx50 (A) and Cx46 (B) levels in total lens homogenates from 2.5-month-old wild-type (+/+), heterozygous (+/Cx50D47A), and homozygous (Cx50D47A/Cx50D47A) mice. (C, D) Bar graphs show the densitometric values of the bands expressed as percentages of the values obtained in wild-type animals (n = 3). Significant differences between wild-type and heterozygous mutant or wild-type and homozygous mutant lenses are indicated by asterisks (P < 0.05). (E, F) Immunoblots show the Cx50 (E) and Cx46 (F) levels in total lens homogenates from 21-day-old wild-type (+/+), heterozygous (+/Cx50D47A), and homozygous (Cx50D47A/Cx50D47A) mice. (G, H) Bar graphs show the densitometric values of the bands expressed as percentages of the values obtained in wild-type animals (n = 3). Significant differences between 21-day-old wild-type and heterozygous mutant or 21-day-old wild-type and homozygous mutant lenses are indicated by asterisks (P < 0.05).

To evaluate whether the abundance of lens fiber cell connexins might explain the decrease in gap junctional coupling between wild-type and Cx50D47A lenses, we measured the levels of Cx46 and Cx50 in total lens homogenates at the same age as gap junction coupling conductance was determined (2.5 months). Levels of Cx46 were decreased to 52% in heterozygotes and to 30% in homozygotes compared to the wild-type values (Figs. 2A−D). Levels of Cx50 were decreased to 11% in heterozygotes and to 1.2% in homozygotes (Figs. 2A−D). To test whether levels of lens fiber connexins were decreased in younger mice, we performed immunoblots of homogenates from 21-day-old lenses. Levels of Cx46 and Cx50 were decreased to 51.1% and 12.9% in heterozygous lenses and to 27.7% and 3.1% in homozygous lenses (Figs. 2E–H).

**Figure 3:**
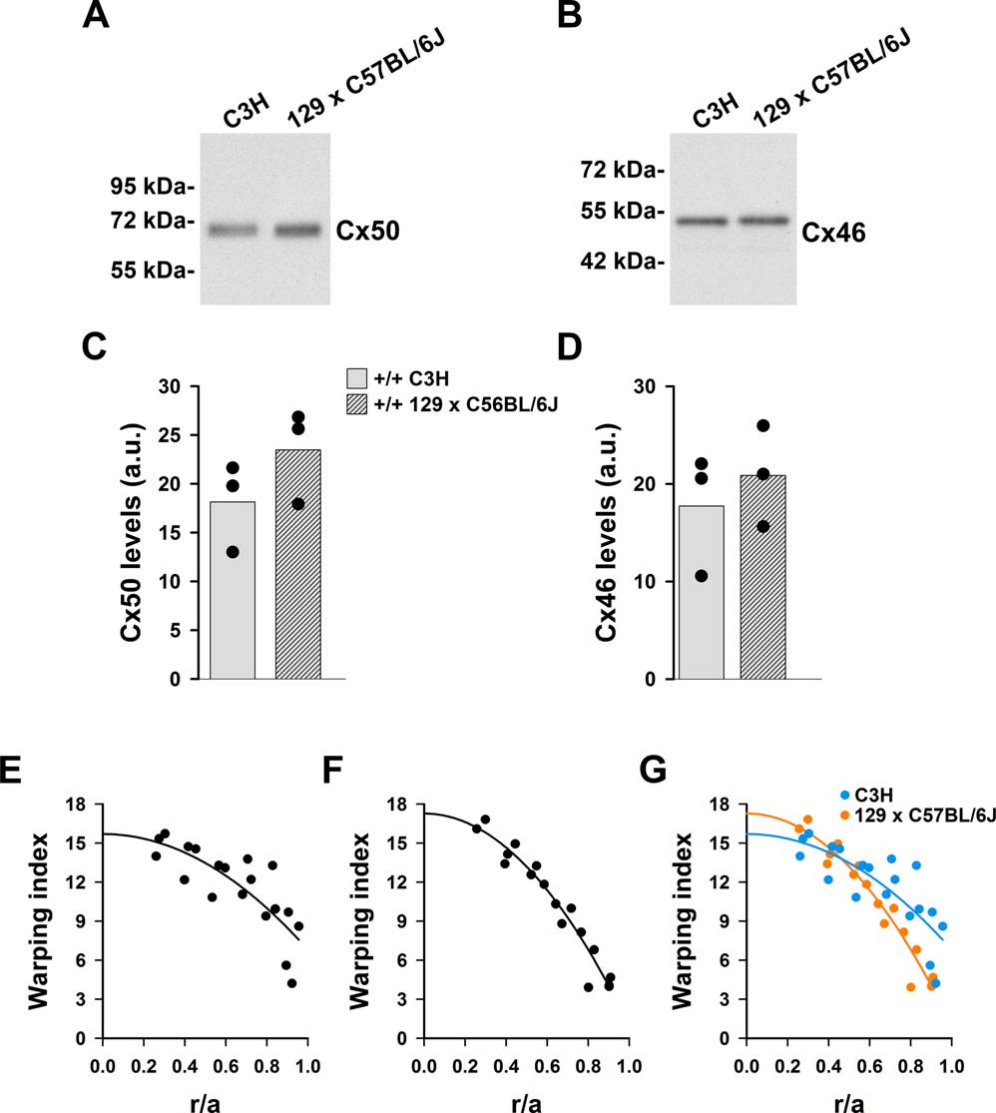
Levels of lens fiber cell connexins are similar in C3H and 129 × C57BL/6J mouse strains. (A, B) Immunoblots show the levels of Cx50 (A) and Cx46 (B) in whole lens homogenates from 1-month-old wild-type mouse lenses from the C3H background and the 129 × C57BL/6J mixed background. (C, D) Bar graphs show the densitometric values in arbitrary units (a.u.) of the Cx50 (C) and Cx46 (D) bands in the C3H and 129 × C57BL/6J backgrounds (n = 3). (E−G) Graphs show the warping index of wild-type lenses from the C3H (E) and 129 × C57BL/6J (F) mice as a function of relative distance from the lens center (r/a) (n = 3). Panel G shows the superposition of the results shown in panels E and F to facilitate comparison between the mouse strains. The curves represent the best-fit of the data to a parabolic function showing only the positive values of x.

The decrease in gap junctional coupling in wild-type lenses from the C3H background compared to the 129 × C57BL/6J mixed background could also be due to reduced levels of connexins; we then compared levels of Cx46 and Cx50 in wild-type lenses from the two mouse strains. We found no significant differences in the levels of fiber cell connexins between the lenses from the C3H strain and those from the 129 × C57BL/6J mixed background (Figs. 3A–D).

### Intracellular Hydrostatic Pressure Is Increased in Mutant Lenses

**Figure 4:**
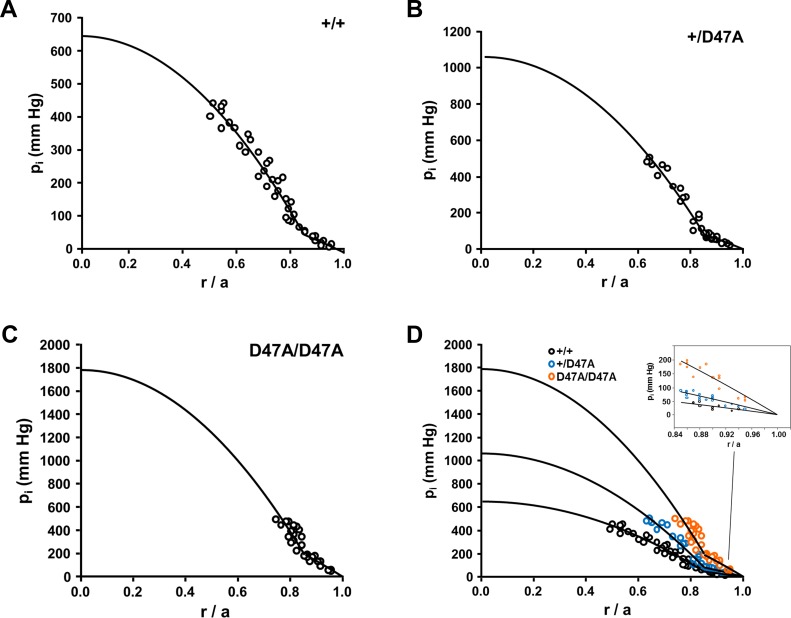
The intracellular hydrostatic pressure is increased in Cx50D47A lenses. (A−C) Graphs show the intracellular hydrostatic pressures (p_i_) measured in lenses from wild-type (A), heterozygous mutant (B), and homozygous mutant (C) mice graphed as a function of normalized radial distance from the lens center (r/a). The curves represent the best fit to the data and their projection to the lens center based on the model described by Gao et al.^22^ (D) Superposition of the results shown in panels A−C to facilitate comparison between the different genotypes. The inset shows an expanded view of the data in the differentiating fiber cells (r/a ≥ 0.85).

Given the important contribution of gap junction coupling conductance to the lens circulation, it was relevant to determine intracellular hydrostatic pressure as an assessment of water flow. In the centers of wild-type C3H lenses, intracellular hydrostatic pressure was 648 mm Hg (i.e., almost twice the value in other mouse strains^10^,^22^–^25^). The central pressure increased from wild-type to heterozygous to homozygous Cx50D47A lenses (inversely to the decrease in gap junction coupling) (Fig. 4; Table 2).

**Table 2:** Intracellular Hydrostatic Pressure in Wild-Type and Cx50D47A Lenses

	**Intracellular Hydrostatic Pressure (mm Hg)**			
Genotype	$\newcommand{\bialpha}{\boldsymbol{\alpha}}\newcommand{\bibeta}{\boldsymbol{\beta}}\newcommand{\bigamma}{\boldsymbol{\gamma}}\newcommand{\bidelta}{\boldsymbol{\delta}}\newcommand{\bivarepsilon}{\boldsymbol{\varepsilon}}\newcommand{\bizeta}{\boldsymbol{\zeta}}\newcommand{\bieta}{\boldsymbol{\eta}}\newcommand{\bitheta}{\boldsymbol{\theta}}\newcommand{\biiota}{\boldsymbol{\iota}}\newcommand{\bikappa}{\boldsymbol{\kappa}}\newcommand{\bilambda}{\boldsymbol{\lambda}}\newcommand{\bimu}{\boldsymbol{\mu}}\newcommand{\binu}{\boldsymbol{\nu}}\newcommand{\bixi}{\boldsymbol{\xi}}\newcommand{\biomicron}{\boldsymbol{\micron}}\newcommand{\bipi}{\boldsymbol{\pi}}\newcommand{\birho}{\boldsymbol{\rho}}\newcommand{\bisigma}{\boldsymbol{\sigma}}\newcommand{\bitau}{\boldsymbol{\tau}}\newcommand{\biupsilon}{\boldsymbol{\upsilon}}\newcommand{\biphi}{\boldsymbol{\phi}}\newcommand{\bichi}{\boldsymbol{\chi}}\newcommand{\bipsi}{\boldsymbol{\psi}}\newcommand{\biomega}{\boldsymbol{\omega}}{p_i}$(0)*	$\newcommand{\bialpha}{\boldsymbol{\alpha}}\newcommand{\bibeta}{\boldsymbol{\beta}}\newcommand{\bigamma}{\boldsymbol{\gamma}}\newcommand{\bidelta}{\boldsymbol{\delta}}\newcommand{\bivarepsilon}{\boldsymbol{\varepsilon}}\newcommand{\bizeta}{\boldsymbol{\zeta}}\newcommand{\bieta}{\boldsymbol{\eta}}\newcommand{\bitheta}{\boldsymbol{\theta}}\newcommand{\biiota}{\boldsymbol{\iota}}\newcommand{\bikappa}{\boldsymbol{\kappa}}\newcommand{\bilambda}{\boldsymbol{\lambda}}\newcommand{\bimu}{\boldsymbol{\mu}}\newcommand{\binu}{\boldsymbol{\nu}}\newcommand{\bixi}{\boldsymbol{\xi}}\newcommand{\biomicron}{\boldsymbol{\micron}}\newcommand{\bipi}{\boldsymbol{\pi}}\newcommand{\birho}{\boldsymbol{\rho}}\newcommand{\bisigma}{\boldsymbol{\sigma}}\newcommand{\bitau}{\boldsymbol{\tau}}\newcommand{\biupsilon}{\boldsymbol{\upsilon}}\newcommand{\biphi}{\boldsymbol{\phi}}\newcommand{\bichi}{\boldsymbol{\chi}}\newcommand{\bipsi}{\boldsymbol{\psi}}\newcommand{\biomega}{\boldsymbol{\omega}}{p_i}$(*b*)	$\newcommand{\bialpha}{\boldsymbol{\alpha}}\newcommand{\bibeta}{\boldsymbol{\beta}}\newcommand{\bigamma}{\boldsymbol{\gamma}}\newcommand{\bidelta}{\boldsymbol{\delta}}\newcommand{\bivarepsilon}{\boldsymbol{\varepsilon}}\newcommand{\bizeta}{\boldsymbol{\zeta}}\newcommand{\bieta}{\boldsymbol{\eta}}\newcommand{\bitheta}{\boldsymbol{\theta}}\newcommand{\biiota}{\boldsymbol{\iota}}\newcommand{\bikappa}{\boldsymbol{\kappa}}\newcommand{\bilambda}{\boldsymbol{\lambda}}\newcommand{\bimu}{\boldsymbol{\mu}}\newcommand{\binu}{\boldsymbol{\nu}}\newcommand{\bixi}{\boldsymbol{\xi}}\newcommand{\biomicron}{\boldsymbol{\micron}}\newcommand{\bipi}{\boldsymbol{\pi}}\newcommand{\birho}{\boldsymbol{\rho}}\newcommand{\bisigma}{\boldsymbol{\sigma}}\newcommand{\bitau}{\boldsymbol{\tau}}\newcommand{\biupsilon}{\boldsymbol{\upsilon}}\newcommand{\biphi}{\boldsymbol{\phi}}\newcommand{\bichi}{\boldsymbol{\chi}}\newcommand{\bipsi}{\boldsymbol{\psi}}\newcommand{\biomega}{\boldsymbol{\omega}}{p_i}$(*a*)	n
+/+	648	46	0	10
+/Cx50D47A	1058	83	0	8
Cx50D47A/Cx50D47A	1784	196	0	8

Table 2 shows the comparison of intracellular hydrostatic pressures, $\newcommand{\bialpha}{\boldsymbol{\alpha}}\newcommand{\bibeta}{\boldsymbol{\beta}}\newcommand{\bigamma}{\boldsymbol{\gamma}}\newcommand{\bidelta}{\boldsymbol{\delta}}\newcommand{\bivarepsilon}{\boldsymbol{\varepsilon}}\newcommand{\bizeta}{\boldsymbol{\zeta}}\newcommand{\bieta}{\boldsymbol{\eta}}\newcommand{\bitheta}{\boldsymbol{\theta}}\newcommand{\biiota}{\boldsymbol{\iota}}\newcommand{\bikappa}{\boldsymbol{\kappa}}\newcommand{\bilambda}{\boldsymbol{\lambda}}\newcommand{\bimu}{\boldsymbol{\mu}}\newcommand{\binu}{\boldsymbol{\nu}}\newcommand{\bixi}{\boldsymbol{\xi}}\newcommand{\biomicron}{\boldsymbol{\micron}}\newcommand{\bipi}{\boldsymbol{\pi}}\newcommand{\birho}{\boldsymbol{\rho}}\newcommand{\bisigma}{\boldsymbol{\sigma}}\newcommand{\bitau}{\boldsymbol{\tau}}\newcommand{\biupsilon}{\boldsymbol{\upsilon}}\newcommand{\biphi}{\boldsymbol{\phi}}\newcommand{\bichi}{\boldsymbol{\chi}}\newcommand{\bipsi}{\boldsymbol{\psi}}\newcommand{\biomega}{\boldsymbol{\omega}}{p_i}$ (mm Hg), from 2-month-old lenses of the three genotypes at the lens center (*p*_*i*_(0)), at the DF-to-MF transition (*p_i_*(*b*)) and at the lens surface (*p_i_*(*a*)). Because the lens centers of the C3H genetic background were very hard and difficult to impale, the values of $\newcommand{\bialpha}{\boldsymbol{\alpha}}\newcommand{\bibeta}{\boldsymbol{\beta}}\newcommand{\bigamma}{\boldsymbol{\gamma}}\newcommand{\bidelta}{\boldsymbol{\delta}}\newcommand{\bivarepsilon}{\boldsymbol{\varepsilon}}\newcommand{\bizeta}{\boldsymbol{\zeta}}\newcommand{\bieta}{\boldsymbol{\eta}}\newcommand{\bitheta}{\boldsymbol{\theta}}\newcommand{\biiota}{\boldsymbol{\iota}}\newcommand{\bikappa}{\boldsymbol{\kappa}}\newcommand{\bilambda}{\boldsymbol{\lambda}}\newcommand{\bimu}{\boldsymbol{\mu}}\newcommand{\binu}{\boldsymbol{\nu}}\newcommand{\bixi}{\boldsymbol{\xi}}\newcommand{\biomicron}{\boldsymbol{\micron}}\newcommand{\bipi}{\boldsymbol{\pi}}\newcommand{\birho}{\boldsymbol{\rho}}\newcommand{\bisigma}{\boldsymbol{\sigma}}\newcommand{\bitau}{\boldsymbol{\tau}}\newcommand{\biupsilon}{\boldsymbol{\upsilon}}\newcommand{\biphi}{\boldsymbol{\phi}}\newcommand{\bichi}{\boldsymbol{\chi}}\newcommand{\bipsi}{\boldsymbol{\psi}}\newcommand{\biomega}{\boldsymbol{\omega}}{p_i}$(0) were estimated by projecting the model fit of peripheral data to the lens center.

The same problem of microelectrode clogging in central fiber cells as described for series resistance measurements above was present in the pressure studies shown in Figure 4. The central pressures were extrapolated from the fit of peripheral data and, therefore, may not be as accurate.

### Lens Refractive Properties Are Altered in Cx50D47A Mice

**Figure 5:**
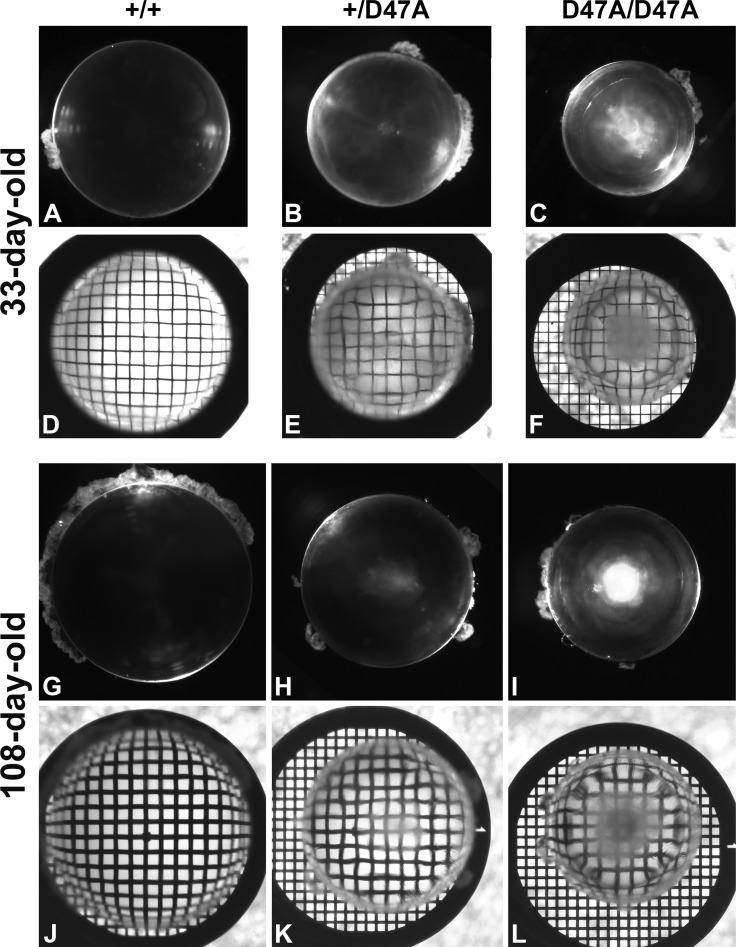
Expression of Cx50D47A alters the refractive properties of the lens. (A−F) Lenses from 33-day-old wild-type (A, D) and mutant heterozygous (B, E) and homozygous (C, F) mice were photographed under dark-field illumination alone (A−C) and on top of a 200 mesh electron microscopy grid (D−F). (G−L) Lenses from 108-day-old wild-type (G, J) and Cx50D47A heterozygous (H, K) and homozygous (I, L) mice were photographed under dark-field illumination alone (G−I) and on top of a 200 mesh electron microscopy grid (J−L). Wild-type lenses produced a barrel deformation of the grid (D, J) that appears distorted in heterozygous (E, K) and homozygous (F, L) lenses.

The intracellular hydrostatic pressure is critical for establishing the refractive index gradient in the lens.^26^,^27^ This conclusion is supported by results from Cx46fs380 mice.^9^,^10^ Therefore, we speculated that the changes in intracellular hydrostatic pressure in Cx50D47A lenses might be accompanied by changes in refractive properties. We tested this hypothesis by photographing lenses through an electron microscopy grid. While 33-day-old wild-type lenses produced a barrel distortion of the grid, heterozygous and homozygous lenses produced complex deformations with a central pincushion (Figs. 5A–F) (Diagrams illustrating barrel and pincushion deformations are shown in figure 5 of Minogue et al.^9^). Older heterozygous and homozygous lenses (108 days old) produced an even more complex distortion of the grid that appeared to result from multiple concentric pincushions of different depths (Figs. 5G−L). It was difficult to focus the grid through heterozygous and homozygous lenses; the cataract obscured the corresponding parts of the grid in homozygous lenses. Consistent with our previous studies,^13^ both heterozygous and homozygous Cx50D47A lenses were smaller than wild-type and had cataracts. The presence of cataracts in these lenses precluded quantification of the warping index, which would have provided a relative measure of refractive changes between wild-type and Cx50D47A lenses. The central location of the cataracts prevented use of laser scan analysis of lens focusing.

Because hydrostatic pressure may affect lens refractive properties, we determined the warping index at different relative distances from the lens center in wild-type C3H and 129 × C57BL/6J mice. The values of warping index showed higher dispersion in the C3H mouse strain (Figs. 3E, 3F). Similar to the graded values of refractive index at different depths within the lens,^28^,^29^ data from both mouse strains could be fit by parabolic functions. However, the best-fit curves for C3H and 129 × C57BL/6J wild-type lenses were different (Fig. 3G).

### Intracellular Calcium Ion Concentrations Are Increased in Mutant Lenses

**Figure 6:**
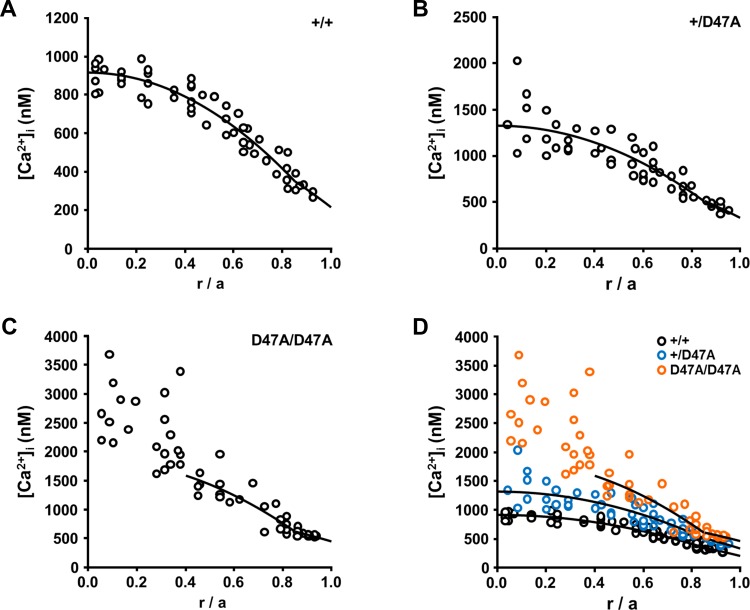
Intracellular calcium ion concentrations are increased in Cx50D47A lenses. (A−C) Plots show the concentrations of intracellular calcium ions ([Ca^2+^]_i_) in wild-type (A) and heterozygous (B) and homozygous (C) lenses as a function of radial distance from the lens center (r; cm), normalized to the lens radius (a; cm). The curves represent the best fit to the data based on the model described in Gao et al.^15^ (D) Superposition of the graphs shown in A, B, and C.

The reductions in gap junction coupling conductance suggested that the gradient of intracellular calcium ion concentrations ([Ca^2+^]_i_) might also be altered. To test this, we measured the [Ca^2+^]_i_ at different depths within the lenses by intracellular injection of the ratiometric calcium indicator dye Fura-2. This gradient increased from wild-type to heterozygous to homozygous Cx50D47A lenses (Fig. 6). The data points closer to the lens center showed progressively increasing scatter in heterozygous and in homozygous lenses as compared with wild-type lenses (Fig. 6). This dispersion of the central data suggests that the control of [Ca^2+^]_i_ may be partially lost in Cx50D47A heterozygous lenses, and there is complete loss of control about halfway into the lens in Cx50D47A homozygous lenses (Fig. 6). The average values of [Ca^2+^]_i_ at different locations within the lens, based on the best-fit of the data shown in Figure 6, are provided in Table 3.

**Table 3:** Intracellular Concentrations of Free Calcium Ions at Different Depths From the Lens Center in Wild-Type and Cx50D47A Lenses

	**[Ca^2+^]_i_ (nM)**			
Genotype	*r* = 0	*r* = *b*	*r* = *a*	*n*
+/+	910	348	210	12
+/Cx50D47A	1321	511	332	12
Cx50D47A/Cx50D47A	?	614	455	12

Average values of [Ca^2+^]_i_ at the lens center ($\newcommand{\bialpha}{\boldsymbol{\alpha}}\newcommand{\bibeta}{\boldsymbol{\beta}}\newcommand{\bigamma}{\boldsymbol{\gamma}}\newcommand{\bidelta}{\boldsymbol{\delta}}\newcommand{\bivarepsilon}{\boldsymbol{\varepsilon}}\newcommand{\bizeta}{\boldsymbol{\zeta}}\newcommand{\bieta}{\boldsymbol{\eta}}\newcommand{\bitheta}{\boldsymbol{\theta}}\newcommand{\biiota}{\boldsymbol{\iota}}\newcommand{\bikappa}{\boldsymbol{\kappa}}\newcommand{\bilambda}{\boldsymbol{\lambda}}\newcommand{\bimu}{\boldsymbol{\mu}}\newcommand{\binu}{\boldsymbol{\nu}}\newcommand{\bixi}{\boldsymbol{\xi}}\newcommand{\biomicron}{\boldsymbol{\micron}}\newcommand{\bipi}{\boldsymbol{\pi}}\newcommand{\birho}{\boldsymbol{\rho}}\newcommand{\bisigma}{\boldsymbol{\sigma}}\newcommand{\bitau}{\boldsymbol{\tau}}\newcommand{\biupsilon}{\boldsymbol{\upsilon}}\newcommand{\biphi}{\boldsymbol{\phi}}\newcommand{\bichi}{\boldsymbol{\chi}}\newcommand{\bipsi}{\boldsymbol{\psi}}\newcommand{\biomega}{\boldsymbol{\omega}}r = 0$) at the DF-to-MF transition ($\newcommand{\bialpha}{\boldsymbol{\alpha}}\newcommand{\bibeta}{\boldsymbol{\beta}}\newcommand{\bigamma}{\boldsymbol{\gamma}}\newcommand{\bidelta}{\boldsymbol{\delta}}\newcommand{\bivarepsilon}{\boldsymbol{\varepsilon}}\newcommand{\bizeta}{\boldsymbol{\zeta}}\newcommand{\bieta}{\boldsymbol{\eta}}\newcommand{\bitheta}{\boldsymbol{\theta}}\newcommand{\biiota}{\boldsymbol{\iota}}\newcommand{\bikappa}{\boldsymbol{\kappa}}\newcommand{\bilambda}{\boldsymbol{\lambda}}\newcommand{\bimu}{\boldsymbol{\mu}}\newcommand{\binu}{\boldsymbol{\nu}}\newcommand{\bixi}{\boldsymbol{\xi}}\newcommand{\biomicron}{\boldsymbol{\micron}}\newcommand{\bipi}{\boldsymbol{\pi}}\newcommand{\birho}{\boldsymbol{\rho}}\newcommand{\bisigma}{\boldsymbol{\sigma}}\newcommand{\bitau}{\boldsymbol{\tau}}\newcommand{\biupsilon}{\boldsymbol{\upsilon}}\newcommand{\biphi}{\boldsymbol{\phi}}\newcommand{\bichi}{\boldsymbol{\chi}}\newcommand{\bipsi}{\boldsymbol{\psi}}\newcommand{\biomega}{\boldsymbol{\omega}}r = b$) and at the lens surface ($\newcommand{\bialpha}{\boldsymbol{\alpha}}\newcommand{\bibeta}{\boldsymbol{\beta}}\newcommand{\bigamma}{\boldsymbol{\gamma}}\newcommand{\bidelta}{\boldsymbol{\delta}}\newcommand{\bivarepsilon}{\boldsymbol{\varepsilon}}\newcommand{\bizeta}{\boldsymbol{\zeta}}\newcommand{\bieta}{\boldsymbol{\eta}}\newcommand{\bitheta}{\boldsymbol{\theta}}\newcommand{\biiota}{\boldsymbol{\iota}}\newcommand{\bikappa}{\boldsymbol{\kappa}}\newcommand{\bilambda}{\boldsymbol{\lambda}}\newcommand{\bimu}{\boldsymbol{\mu}}\newcommand{\binu}{\boldsymbol{\nu}}\newcommand{\bixi}{\boldsymbol{\xi}}\newcommand{\biomicron}{\boldsymbol{\micron}}\newcommand{\bipi}{\boldsymbol{\pi}}\newcommand{\birho}{\boldsymbol{\rho}}\newcommand{\bisigma}{\boldsymbol{\sigma}}\newcommand{\bitau}{\boldsymbol{\tau}}\newcommand{\biupsilon}{\boldsymbol{\upsilon}}\newcommand{\biphi}{\boldsymbol{\phi}}\newcommand{\bichi}{\boldsymbol{\chi}}\newcommand{\bipsi}{\boldsymbol{\psi}}\newcommand{\biomega}{\boldsymbol{\omega}}r = a$). The [Ca^2+^]_i_ at the center (*r* = 0) for homozygous Cx50D47A lenses has been marked with a “?” because the model fit of data at *r*/$\newcommand{\bialpha}{\boldsymbol{\alpha}}\newcommand{\bibeta}{\boldsymbol{\beta}}\newcommand{\bigamma}{\boldsymbol{\gamma}}\newcommand{\bidelta}{\boldsymbol{\delta}}\newcommand{\bivarepsilon}{\boldsymbol{\varepsilon}}\newcommand{\bizeta}{\boldsymbol{\zeta}}\newcommand{\bieta}{\boldsymbol{\eta}}\newcommand{\bitheta}{\boldsymbol{\theta}}\newcommand{\biiota}{\boldsymbol{\iota}}\newcommand{\bikappa}{\boldsymbol{\kappa}}\newcommand{\bilambda}{\boldsymbol{\lambda}}\newcommand{\bimu}{\boldsymbol{\mu}}\newcommand{\binu}{\boldsymbol{\nu}}\newcommand{\bixi}{\boldsymbol{\xi}}\newcommand{\biomicron}{\boldsymbol{\micron}}\newcommand{\bipi}{\boldsymbol{\pi}}\newcommand{\birho}{\boldsymbol{\rho}}\newcommand{\bisigma}{\boldsymbol{\sigma}}\newcommand{\bitau}{\boldsymbol{\tau}}\newcommand{\biupsilon}{\boldsymbol{\upsilon}}\newcommand{\biphi}{\boldsymbol{\phi}}\newcommand{\bichi}{\boldsymbol{\chi}}\newcommand{\bipsi}{\boldsymbol{\psi}}\newcommand{\biomega}{\boldsymbol{\omega}}a$ ≥ 0.4 suggested a functional circulation would generate about 2000 nM, but essentially all the data were above this value.

### Cx50D47A Lenses Contain Calcium Precipitates

**Figure 7:**
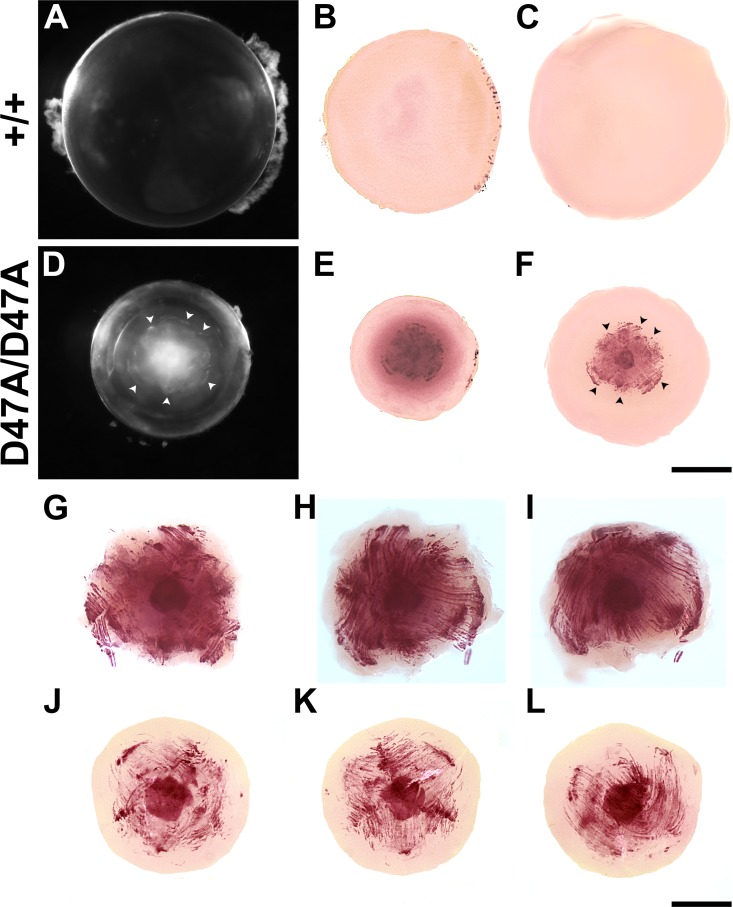
Cx50D47A lenses show an intricate pattern of Alizarin red staining. (A, D) Photographs of 21-day-old wild-type (+/+) and Cx50D47A homozygous (D47A/D47A) lenses using dark-field illumination. (B, C, E, F) Images showing the same lenses from wild-type (B, C) and Cx50D47A homozygotes (E, F) after whole-mount staining with Alizarin red before (B, E) and after decapsulation (C, F). Arrowheads point to the Alizarin red-stained precipitates that can be mapped to the cataract outline. (G−L) Images show the Alizarin red staining of the central region of two different 21-day-old Cx50D47A homozygous lenses photographed from different angles. Panels G−I correspond to the central region of the lens shown in panels D−F. Scale bars: 591 μm for panels A−F and 297 μm for panels G−L.

The [Ca^2+^]_i_ that we measured in Cx50D47A lenses exceeds the K*_sp_* for several calcium salts. To test whether these lenses contained calcium precipitates, we stained wild-type and homozygous lenses with Alizarin red. Wild-type lenses showed unremarkable staining with Alizarin red (Figs. 7A−C). In contrast, we detected extensive staining in the central region of the homozygous lens (Figs. 7D−L). The very center was uniformly and intensely stained (Figs. 7E−L). It was surrounded by a region with an intricate linear pattern of staining, consistent with the morphology and orientation of lens fibers (Figs. 7F−L). More peripherally, we detected a halo of more diffuse staining, suggesting the presence of microprecipitates in this region (Fig. 7E); this staining was lost after further destaining and decapsulation of the lenses (Fig. 7F). The overall pattern of fiber-like staining appeared to correspond to the boundary of the central cataract (compare Figs. 7D and 7F as indicated by the arrowheads); shrinkage during processing hampers direct visual comparison between the darkfield images and the Alizarin red-stained lenses.

## Discussion

We have previously shown that Cx50D47A lenses have cataracts and altered levels of several proteins and are smaller than wild-type lenses.^13^,^18^ In this study, we further characterized functional properties and components of the lens circulation in these lenses. We found that Cx50D47A lenses had disruptions in the lens circulation, leading to calcium accumulation, and formation of calcium precipitates.

### Effects of Cx50D47A Expression on the Lens Circulation

Cx50D47A lenses had decreased gap junctional conductance compared to wild-type lenses (Fig. 1). The decreases in gap junctional conductance likely result from reduced levels of Cx50 and Cx46 (Figs. 2A−D). The relations between connexin levels and conductances in differentiating and mature lens fibers may indicate the relative contributions of Cx46 and Cx50 in those regions. At the age that coupling was studied, levels of Cx46 were 52% of wild-type values in heterozygous Cx50D47A lenses, while Cx50 was reduced to 11%, suggesting that the remaining coupling in differentiating (49%) and mature fibers (24%) is provided predominantly by Cx46. In homozygous Cx50D47A lenses, Cx50 levels approached zero and the remaining conductance in differentiating fibers (29%) was similar to the Cx46 levels (30% of wild-type); therefore, gap junctional coupling must be almost exclusively dependent on Cx46.

Lenses of heterozygous and homozygous Cx50D47A mice had increased gradients of hydrostatic pressure that were qualitatively predictable from the lens circulation model.^22^ In this model, the intracellular outflow of fluid and ionic current is mediated by fiber cell gap junctions; therefore, the effective intracellular hydraulic and electrical conductivities should be proportional.^22^ However, in our data, the increase in hydrostatic pressure from wild-type to heterozygote to homozygote was somewhat less than the reduction in gap junction coupling conductance. These results suggest that expression of Cx50D47A also had some indirect effects that reduced water flow. Changes in refractive properties in Cx50D47A lenses may also be related to the increased gradients of hydrostatic pressure, because the gradient of refractive index in the lens is a function of the protein concentration gradient.

In Cx50D47A lenses, the intracellular hydrostatic pressure at the lens surface was zero (as in wild-type lenses). It is also zero in Cx46fs380 lenses, heterozygous AQP0-null lenses, heterozygous Cx46-null lenses, glutathione peroxidase 1-null lenses, and Cx46KI lenses (in which Cx50 is replaced by Cx46), but it is positive in lenses from mice with deletion of PTEN.^10^,^22^–^24^

### Expressing a Cx50 Mutant Is Not the Same as Deleting Cx50

Our studies show that gap junctional coupling is more severely decreased in Cx50D47A lenses than in Cx50-null lenses.^30^ As shown in Table 1, both G_DF_ and G_MF_ were lower in heterozygous and homozygous Cx50D47A lenses (as compared to wild-type values) than in Cx50^+/−^ or Cx50^−/−^ lenses. The increased severity of the reductions of gap junctional conductance in Cx50D47A lenses likely reflects reductions of both Cx46 and Cx50 levels, unlike the Cx50-null lenses in which deletion of Cx50 does not alter Cx46 abundance.^30^ The reduced conductances in Cx50D47A lenses (G_DF_ of 49% and G_MF_ of 24% in heterozygotes and G_DF_ of 29% and G_MF_ of 4% in homozygotes) suggest that this is more than a simple loss-of-function mutant. The data imply that Cx50D47A does not contribute to gap junctional coupling conductance, and that the mutant also inhibits the co-expressed wild-type Cx50 (in heterozygotes) and Cx46 (in both heterozygotes and homozygotes). This interpretation supports our previous data suggesting that Cx50D47A impairs the trafficking and/or stability of wild-type Cx50 and Cx46 in the lens.^13^ Similarly, Cx46fs380, a Cx46 mutant, may inhibit its own trafficking to the plasma membrane and that of the co-expressed wild-type Cx46 and Cx50 in the lens.^31^ Thus, reducing the levels of co-expressed wild-type connexins may be a general mechanism by which expression of mutant lens fiber connexins leads to dominantly inherited cataracts.

### Components of the Internal Circulation Differ Among Wild-Type Lenses From Different Mouse Strains

To our surprise, the gap junctional coupling conductance of the wild-type mouse lenses in our current study (C3H strain) was significantly lower than that previously determined in wild-type lenses from other genetic backgrounds (including C57BL/6J and 129/Sv × C57BL/6J).^9^,^25^,^30^,^32^ In wild-type C3H lenses, we found G_DF_ = 0.41 S/cm^2^ and G_MF_ = 0.25 S/cm^2^ (Table 1). In other strains, G_DF_ is ∼ 1.0 S/cm^2^ and G_MF_ is ∼ 0.4–0.5 S/cm^2^ (see Table 1 for the values in the 129/Sv × C57BL/6J strain, as determined by Baldo et al.).^30^ Thus, in C3H lenses, gap junctional conductance of differentiating fibers is less than half that in this region in other strains; it becomes further decreased in mature fibers, but the relative decrement is less than in other strains. The decrease in gap junctional conductance in C3H lenses cannot result from differences in connexin levels, because Cx46 and Cx50 levels were not significantly different in lenses of wild-type C3H and 129 × C57BL/6J mice (Figs. 3A−D). The decrease in gap junctional conductance in the C3H strain may reflect a reduced open probability of the gap junction channels.

The differences in gap junctional coupling between strains may explain some other physiological differences: Intracellular hydrostatic pressure in the centers of wild-type C3H lenses was almost twice that in wild-type lenses from other mouse strains^10^,^22^–^25^ (consistent with the measured gap junction coupling of about half the values in other mouse strains). The increase in intracellular hydrostatic pressure may have affected the gradient of refractive index of wild-type C3H lenses, because the changes in warping index were different than in lenses from the 129 × C57BL/6J mixed background. However, the hydrostatic pressure may not be the only contributing factor. The [Ca^2+^]_i_ gradient (i.e., the difference in [Ca^2+^]_i_ between the lens center and surface) was elevated relative to that measured in lenses from 129/Sv × C57BL/6J mice: 700 nM in wild-type C3H lenses (this study) versus 400 nM in 129/Sv × C57BL/6J wild-type lenses.^15^

In addition, we found that C3H wild-type lenses were larger (i.e., greater radii) than 129/Sv × C57BL/6J wild-type lenses (Table 1). This difference cannot result from age differences, since the studies of both mouse strains were done at ∼ 2 months, and the lenses only grow by ∼ 5% between 2 and 3 months of age.^25^

### Calcium Ion Accumulation in Cx50D47A Lenses and Formation of Calcium Precipitates

Cx50D47A lenses had increased [Ca^2+^]_i_ compared to wild-type C3H lenses. This change likely resulted in part from the decrease in gap junctional coupling. The increase of [Ca^2+^]_i_ in homozygous Cx50D47A lenses is greater than that determined in Cx46^−/−^ lenses^15^ or in homozygous Cx46fs380 lenses.^10^ Indeed, the values of [Ca^2+^]_i_ in the center of homozygous Cx50D47A lenses were similar to the highest values of [Ca^2+^]_i_ determined in 14-month-old wild-type lenses at r/a = 0.2.^25^ The greater increase in [Ca^2+^]_i_ in Cx50D47A lenses may explain why the cataracts are more severe and detectable at a younger age than the cataracts that develop in Cx46^−/−^ and Cx46fs380 lenses.

In a lens with a functional circulation, entry of calcium ions across central fiber cell membranes is balanced by their exit through gap junctions to flow to the lens surface for membrane based extrusion. If that balance is lost, calcium will accumulate in central fibers. This process seems to be occurring in Cx50D47A lenses. In heterozygotes, the model predicted a [Ca^2+^]_i_ of ∼1300 nM at the lens center, but the data were scattered and the maximum value measured was ∼2000 nM. In homozygous lenses, the model suggested that a functional circulation would generate a [Ca^2+^]_i_ at the lens center of ∼2000 nM, but essentially all the data were above this value, with the greatest value being 3700 nM. The [Ca^2+^]_i_ determined in heterozygous and homozygous Cx50D47A lenses exceeds the K*_sp_* values for many calcium salts. It is likely that a significant proportion of the Ca^2+^ in mature fiber cells of Cx50D47A lenses is in the form of precipitates. The intense and massive Alizarin red staining of the centers of Cx50D47A homozygous lenses supports this hypothesis.

Cataract formation associated with calcium precipitation may be a common process. Human cataractous lenses have previously been reported to contain increased [Ca^2+^]_i_ and calcium oxalate or calcium carbonate crystals.^33^–^36^ In homozygous Cx50D47A lenses, the Alizarin red staining localized to the same region occupied by the cataract, and its overall pattern of staining resembled the cataract morphology. We previously observed that cataracts in mice carrying the Cx46fs380 mutant also stain with Alizarin red.^10^

Taken together, our results suggest a pathologic sequence of events for individuals carrying cataract-associated connexin mutant genes. Reduced connexin levels lead to decreased gap junctional conductance, disrupting the internal circulation of the lens, and leading to accumulation of Ca^2+^ that precipitates and forms lens opacities.
